# PSD-95 in the anterior cingulate cortex contributes to neuropathic pain by interdependent activation with NR2B

**DOI:** 10.1038/s41598-022-21488-7

**Published:** 2022-10-12

**Authors:** Ang Li, Chang-Jun Huang, Kai-Peng Gu, Yan Huang, Ya-Qin Huang, Hui Zhang, Jia-Piao Lin, Yu-Fan Liu, Yan Yang, Yong-Xing Yao

**Affiliations:** 1 Department of Anesthesia, First Affiliated Hospital, Zhejiang University School of Medicine, 79 Qingchun Road, Hangzhou, 310003 China; 2 Department of Anesthesia, First People’s Hospital of Linping District, Hangzhou, China; 3 Department of Neurobiology, Sir Run Run Shaw Hospital, Zhejiang University School of Medicine, Hangzhou, China

**Keywords:** Neuroscience, Neurology

## Abstract

Studies suggest that the scaffolding protein, postsynaptic density protein-95 (PSD-95), is involved in multiple neurological dysfunctions. However, the role of PSD-95 in the anterior cingulate cortex (ACC) in neuropathic pain (NP) has not been investigated. The current study addressed the role of PSD-95 in the ACC in NP and its modulating profile with NMDA receptor subunit 2B (NR2B). The NP model was established by chronic constriction injury (CCI) of the sciatic nerve, and mechanical and thermal tests were used to evaluate behavioral hyperalgesia. Protein expression and distribution were evaluated using immunohistochemistry and western blotting. The results showed that PSD-95 and NR2B were co-localized in neurons in the ACC. After CCI, both PSD-95 and NR2B were upregulated in the ACC. Inhibiting NR2B with Ro 25-6981 attenuated pain hypersensitivity and decreased the over-expression of PSD-95 induced by CCI. Furthermore, intra-ACC administration of PSD-95 antisense oligonucleotide not only attenuated pain hypersensitivity but also downregulated the NR2B level and the phosphorylation of cyclic AMP response element-binding protein. These results demonstrated that PSD-95 in the ACC contributes to NP by interdependent activation of NR2B.

## Introduction

Neuropathic pain (NP), resulting from lesions or diseases of the somatosensory system, is characterized by aberrant spontaneous pain, allodynia, and hyperalgesia^1,2^. Because of the obscure mechanisms underlying NP, current therapeutic measures are often ineffective^3,4^. Studies have suggested that central and peripheral sensitization are the fundamental factors involved in NP^5–7^. The anterior cingulate cortex (ACC), on the inner side of the cerebral hemispheres, is the frontal part of the cingulate cortex that resembles a “collar” surrounding the frontal part of the corpus callosum, and is heterogeneous with high intrinsic plasticity. The ACC appears to play roles in a wide variety of autonomic functions, such as regulating blood pressure and heart rate^8,9^. However, the role of the ACC in pain processing is largely unknown. The ACC is largely known to be involved in pain emotion modulation, but recent evidence suggests that it is involved in pain-related central sensitization, which contributes to behavioral hypersensitivity in pain perception^10–12^. However, compared to the spinal cord, far less is known about the ACC, although new insights into NP manipulation and intervention are expected in supraspinal areas^13,14^.

Postsynaptic density protein-95 (PSD-95), a synaptic scaffolding molecule in the postsynaptic density, binds to the C-terminus of the N-methyl D-aspartate receptor 2B subunit (NR2B) through the N-terminal PDZ domain^15^. Cortical PSD-95 is critical in learning, decision making, and cortex plasticity^16–18^, and spinal PSD-95 is involved in neuropathic pain^19,20^. On the other hand, the activation of NR2B has been proved to play important roles in the development of central sensitization and NP^21,22^. However, the role of PSD-95 in the ACC and its modulating profile with NR2B during NP has not been investigated. Since the interaction of PSD-95 with NR2B is essential in the development of central sensitization, resolving their interaction pathway and downstream signaling is crucial for both the development and maintenance of NP^23,24^.

Cyclic AMP response element-binding protein (CREB) is a constitutive transcription factor that binds to the cAMP-responsive element sequence in the promoter region and plays a substantial role in synaptic plasticity and neurological diseases^25^. It has been reported that CREB in the ACC is involved in pain modulation as a downstream target of PSD-95^26^. However, the relationship between CREB and PSD-95 in the ACC in NP needs to be further elucidated.

In the current study, we addressed the role of PSD-95 in the ACC and its relationship with NR2B in a rat chronic constriction injury (CCI) model using antisense oligonucleotides (AS-ODN) and pharmacological strategies. We aimed to elucidate the synaptic mechanisms underlying peripheral nerve injury-induced NP and identify potential targets for intervention.

## Results

### The cellular distribution of PSD-95 and NR2B in the ACC

**Figure 1:**
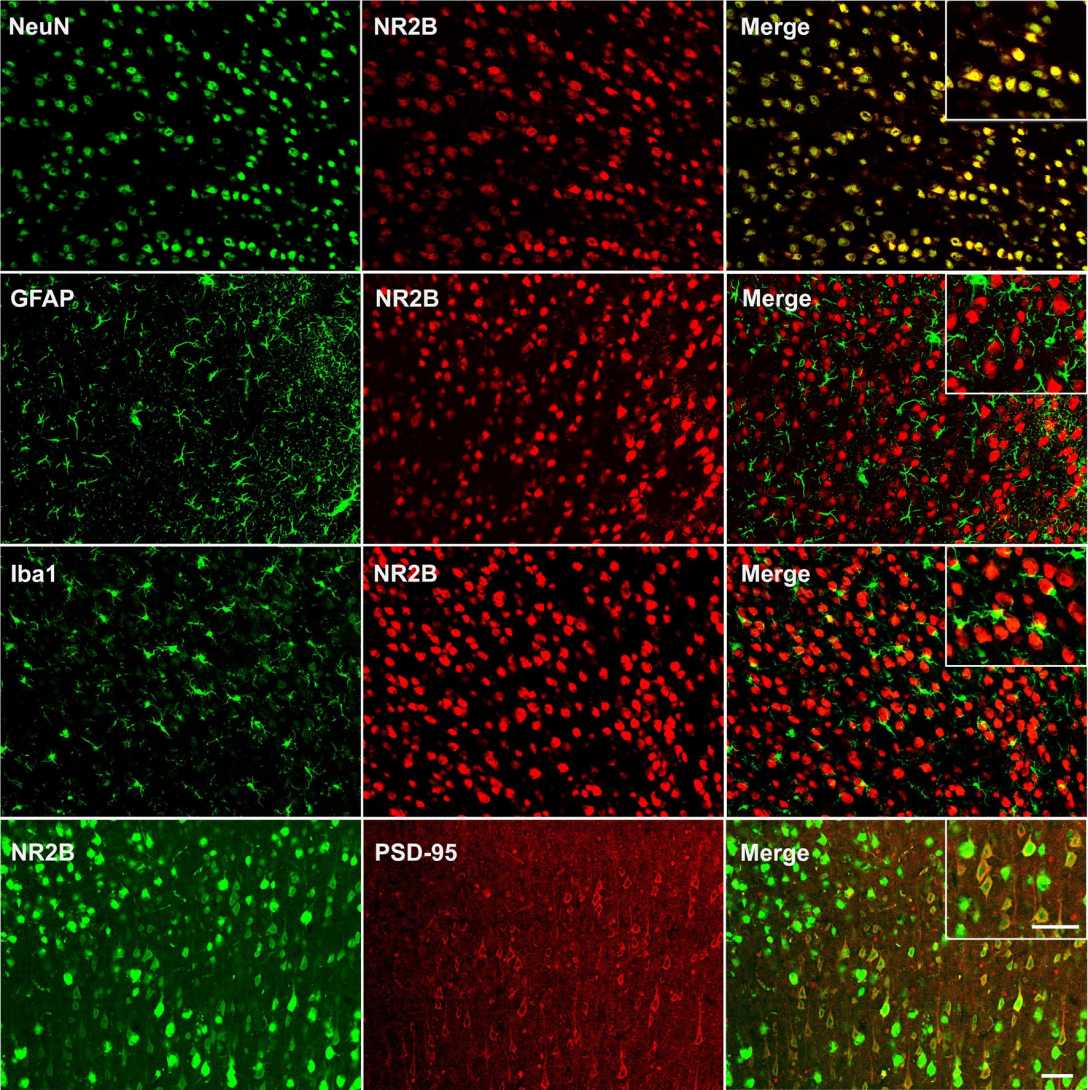
Cellular distribution of NR2B and PSD-95 in the anterior cingulate cortex. Double immunofluorescence staining shows colocalization of NR2B with NeuN and PSD-95, but not with GFAP or Iba1. Scale bar = 100 μm. NR2B: NMDA receptor subunit 2B; NeuN: neuron-specific nuclear protein; GFAP: glial fibrillary acidic protein; Iba1: ionized calcium-binding adaptor molecule-1; PSD-95: postsynaptic density protein-95.

Immunofluorescence experiments were conducted to explore the expression profiles of PSD-95 and NR2B in the ACC. We performed double immunofluorescence for NR2B with neuron-specific nuclear protein (NeuN), glial fibrillary acidic protein (GFAP), ionized calcium-binding adaptor molecule-1 (Iba1), and PSD-95. The results showed that NR2B was co-localized well with NeuN and PSD-95, not with GFAP or Iba1 (Fig. 1).

### CCI-induced behavioral hyperalgesia, upregulation of PSD-95/NR2B, and activation of CREB in the ACC

**Figure 2:**
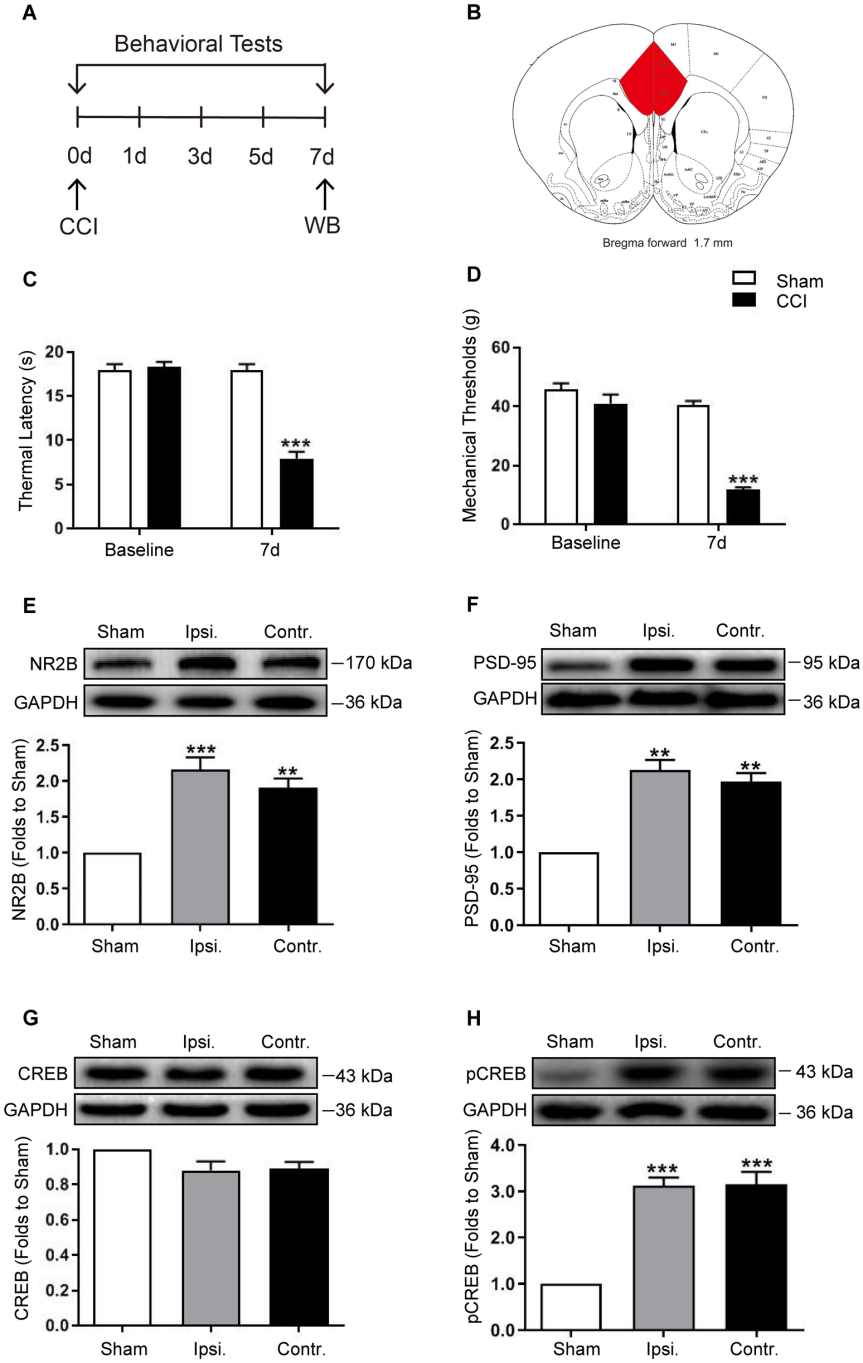
CCI-induced behavioral hypersensitivity, NR2B/PSD-95 upregulation, and CREB activation in the ACC. (**A**) Schematic diagram of the experiment. (**B**) The anatomical boundaries used for ACC tissue harvest. In comparison with the sham group, the CCI group showed significantly lower thermal latency (**C**) and mechanical threshold (**D**) on the 7th day after surgery (***P < 0.001, independent *t-*test,* n* = 6). Western blot showing that compared to that in the sham group, the expression of NR2B (**E**) and PSD-95 (**F**) in the ACC was bilaterally increased in the CCI group (***P < 0.001, **P < 0.01, vs. Sham, one-way ANOVA,* n* = 4); while the total CREB (**G**) did not change, the level of pCREB (**H**) was bilaterally increased in the CCI group (***P < 0.001, vs. Sham, one-way ANOVA,* n* = 4). GAPDH was loading control and error bars represent standard error of the mean (SEM). Original images of blots are available in supplementary Fig. S2. ACC: anterior cingulate cortex; CCI: chronic constriction injury; Ipsi: Ipsilateral; Contr: Contralateral; NR2B: NMDA receptor subunit 2B; CREB: Cyclic AMP response element-binding protein; pCREB: phosphorylated CREB; GAPDH: glyceraldehyde-3-phosphate dehydrogenase.

CCI was adopted to induce NP. Behavioral hyperalgesia was determined by thermal latency (TL) and mechanical threshold (MT) after the CCI (Fig. 2A). For protein expression, the ACC was harvested for western blot analysis (Fig. 2B). No significant difference was observed in the baseline pain behavior between the sham and CCI groups. Seven days after surgery, the TL and MT in the CCI group were significantly lower than those in the sham group (Fig. 2C, D;* P* < 0.001, independent *t-*test, *n* = 6). These results suggest that CCI successfully induced behavioral hyperalgesia. To explore whether NR2B and PSD-95 are involved in the component of pain sensation, the expressions of NR2B and PSD-95 were detected in the ACC. The results showed that consistent with the behavioral hyperalgesia, the expressions of NR2B and PSD-95 were significantly higher than those in the sham group on day 7 after CCI (Fig. 2E, F;* P* < 0.01 or 0.001, one-way analysis of variance [ANOVA], *n* = 4). These data demonstrated that after CCI, NR2B and PSD-95 were activated in the ACC.

To explore the potential mechanisms involved, we detected the activation of CREB in the ACC after CCI. Western blotting showed that, although the total CREB was not altered, phosphorylated CREB was increased significantly in the CCI group (Fig. 2G, H;* P* < 0.001, one-way ANOVA, *n* = 4). These results demonstrated that CREB was activated in the ACC.

### Ro 25-6981 attenuated hyperalgesia and suppressed PSD-95 over-expression

**Figure 3:**
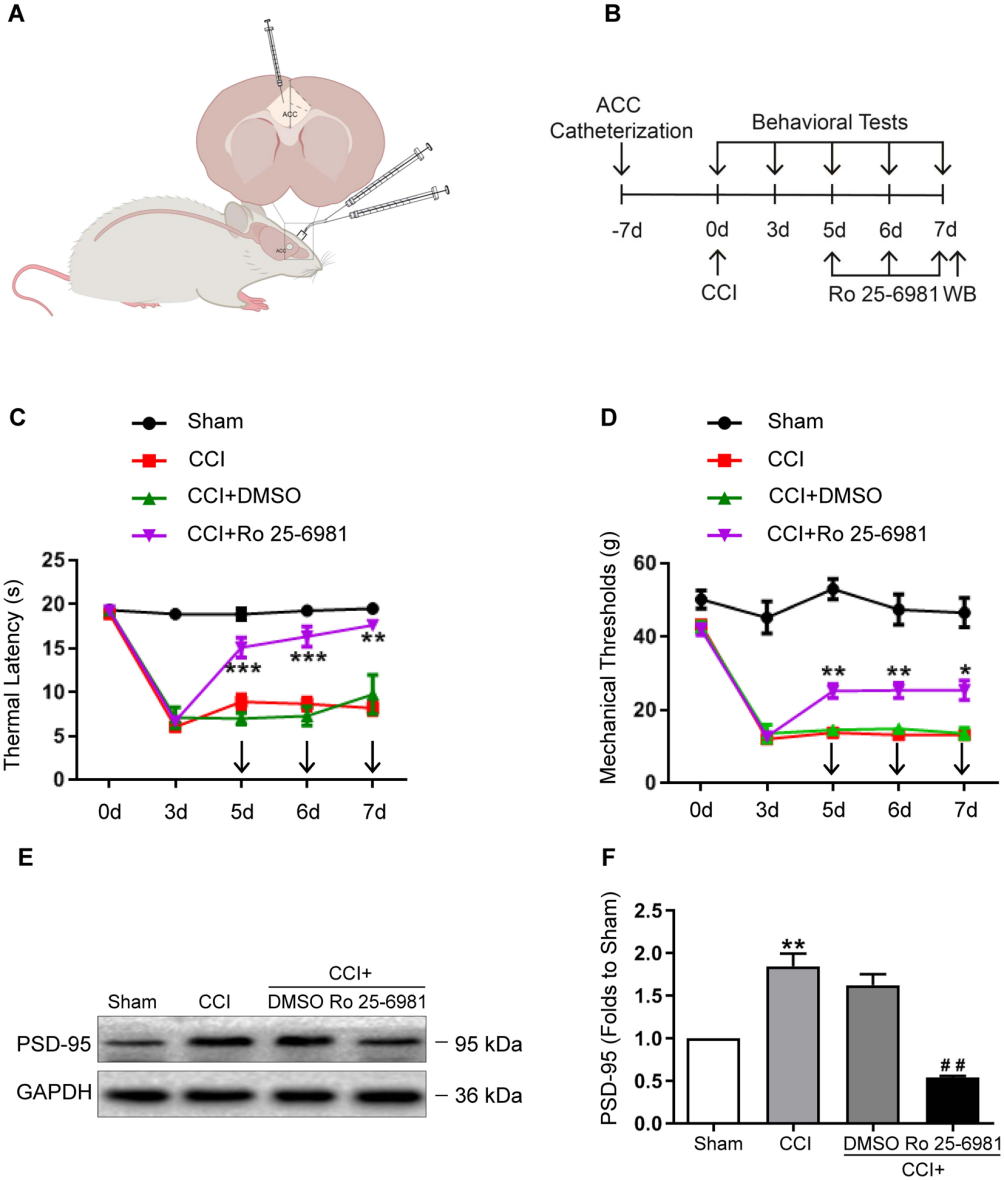
Ro 25-6981 attenuated behavioral hypersensitivity and inhibited the PSD-95 upregulation induced by CCI. (**A**, **B**) Schematic diagram of the experiments. In comparison with DMSO, Ro 25-6981 increased the thermal latency (**C**) and mechanical threshold (**D**) (*P < 0.05, **P < 0.01, ***P < 0.001, vs. CCI + DMSO, two-way ANOVA,* n* = 6). (**E**, **F**) Ro 25-6981 suppressed PSD-95 over-expression induced by CCI (**P < 0.01, vs. sham; ^##^*P* < 0.01, vs. CCI + DMSO, one-way ANOVA, *n* = 3). GAPDH was loading control and error bars represent standard error of the mean (SEM). Original images of blots are available in supplementary Fig. S4. CCI: chronic constriction injury; DMSO: dimethyl sulfoxide; PSD-95: postsynaptic density protein-95; GAPDH: glyceraldehyde-3-phosphate dehydrogenase.

To explore the role of NR2B in the development of NP and PSD-95 activation in the ACC, Ro 25-6981 was injected into the ACC on days 5, 6, and 7 following CCI. TL and MT were determined 2 h after injection (Fig. 3A, B). The behavioral results showed that, compared to the dimethyl sulfoxide (DMSO) group, Ro 25-6981 significantly increased the TL and MT from the first to the third application (Fig. 3C, D;* P* < 0.05, 0.01 or 0.001, two-way ANOVA, *n* = 6). In addition, western blotting results showed that injections of Ro 25-6981 downregulated the over-expression of PSD-95 induced by CCI (Fig. 3E, F;* P* < 0.01, one-way ANOVA, *n* = 3). These findings indicate that NR2B in the ACC plays an essential role in the development of NP, while PSD-95 might be a downstream target in pain manipulation.

### PSD-95 AS-ODN reversed hyperalgesia and inhibited the PSD-95/NR2B over-expression induced by CCI

**Figure 4:**
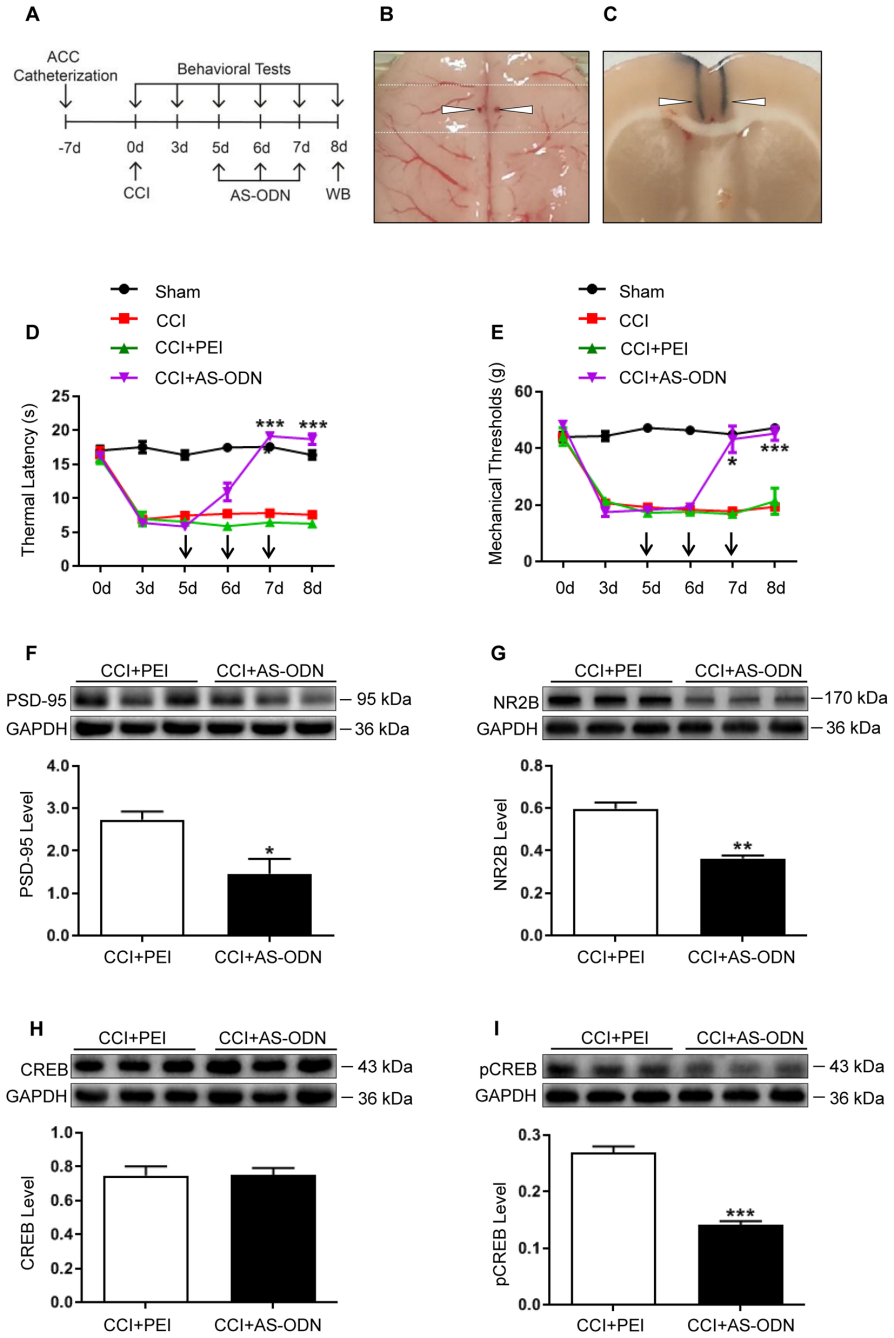
PSD-95 AS-ODN attenuated behavioral hypersensitivity, inhibited the PSD-95/NR2B over-expression and CREB activation induced by CCI. (**A**) Schematic diagram of the experiment. (**B**, **C**) The location of ACC catheterization and microinjection. (**D**, **E**) In comparison with the PEI, the thermal latency and mechanical threshold were significantly higher in the AS-ODN group (*P < 0.05, ***P < 0.001, vs. CCI + PEI, two-way ANOVA,* n* = 6). (**F**) Western blot showed that the expression of PSD-95 in the AS-ODN group was significantly lower than that in the PEI group (*P < 0.05, vs. CCI + PEI, independent *t-*test,* n* = 3). (**G**) The expression of NR2B in the AS-ODN group was significantly lower than that in the PEI group (**P < 0.01, vs. CCI + PEI, independent *t-*test,* n* = 3). (**H**, **I**) The expression level of CREB was not altered significantly. The phosphorylated CREB was significantly lower than that in the PEI control group (***P < 0.001, vs. CCI + PEI, independent *t-*test,* n* = 3). GAPDH was loading control, and error bars represent standard error of the mean (SEM). Original images of blots are available in supplementary Fig. S3. ACC: anterior cingulate cortex; CCI: chronic constriction injury; PEI: polyethyleneimine; AS-ODN: antisense oligonucleotide; PSD-95: postsynaptic density protein-95; NR2B: NMDA receptor subunit 2B; CREB: cyclic AMP response element-binding protein; pCREB: phosphorylated CREB; GAPDH: glyceraldehyde-3-phosphate dehydrogenase.

To further investigate the role of PSD-95 in CCI-induced pain hypersensitivity, AS-ODN was injected into the ACC on days 5, 6, and 7 after the CCI. The TL and MT were measured 2 h after every ACC administration and 1 day after the last administration, according to a pilot experiment (Fig. 4A–C). The results showed that both the TL and MT were significantly increased in the AS-ODN group on the last administration day and the following day (Fig. 4D, E;* P* < 0.05 or 0.001, two-way ANOVA, *n* = 6). Western blotting analysis showed that the expression level of PSD-95 in the AS-ODN group was significantly lower than that in the control group (Fig. 4F;* P* < 0.05, one-way ANOVA, *n* = 3). The AS-ODN also significantly reduced the expression of NR2B (Fig. 4G;* P* < 0.01, one-way ANOVA, *n* = 3). Although the level of total CREB did not change, the phosphorylated CREB was significantly downregulated by PSD-95 AS-ODN (Fig. 4H, I;* P* < 0.001, one-way ANOVA, *n* = 3). These results suggest that consecutive application of PSD-95 AS-ODN not only attenuated the behavioral hypersensitivity, but also inhibited the over-expression of NR2B and phosphorylation of CREB induced by CCI, indicating that in the ACC, PSD-95 plays a critical role in pain hyperalgesia, and CREB may be a potential downstream molecular target of PSD-95 in NP.

### The effect of ACC injection on locomotive ability

**Figure 5:**
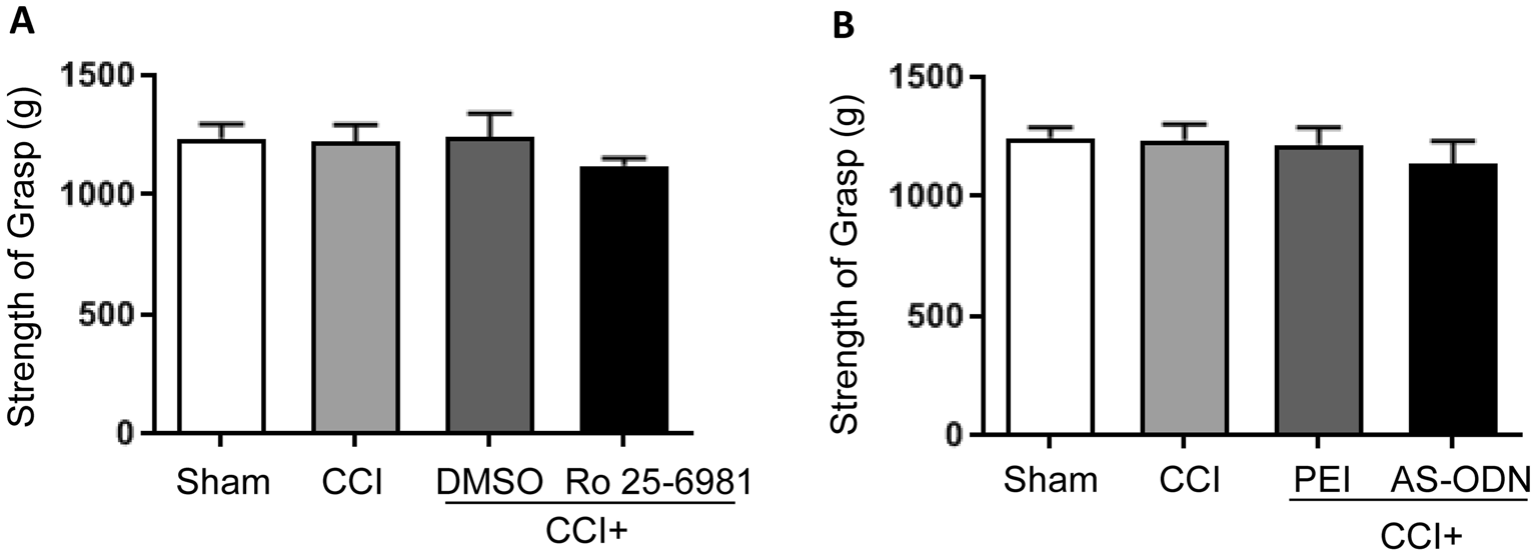
The effect of ACC catheterization and manipulation on locomotive abilities of the front paws. (**A**, **B**) The strength of grasp of the front paws showed no significant differences, compared to the control groups (*n* = 6). AS-ODN: antisense oligonucleotide; ACC: anterior cingulate cortex.

To rule out locomotive ability impairment after ACC catheterization and manipulation, the myodynamia of the front paws was tested at the corresponding time after CCI. Compared to the control groups, no significant differences were observed in the Ro 25-6981 (Fig. 5A; *n* = 6) or AS-ODN (Fig. 5B; *n* = 6) groups. These data demonstrated that neither ACC catheterization nor microinjection impaired the locomotive ability of rats.

## Discussion

In the present study, we investigated the distribution of PSD-95 and NR2B in the ACC and their potential roles in NP. We found that NR2B was expressed in neurons, and PSD-95 co-localized well with NR2B. After CCI, expressions of both NR2B and PSD-95 were increased, consistent with behavioral hypersensitivity. Functional inhibition of NR2B or knockdown of PSD-95 expression attenuated CCI-induced hyperalgesia, suggesting that NR2B and PSD-95 in the ACC play critical roles in the development of NP. More importantly, while Ro 25-6981 suppressed the over-expression of PSD-95, PSD-95 AS-ODN also suppressed the over-expression of NR2B, implying that NR2B and PSD-95 were interdependently activated in the process of NP. In addition, the activation of CREB was also inhibited after PSD-95 knockdown, indicating that CREB may be a downstream target of PSD-95 in the ACC.

Although the ACC is recognized to play a critical role in the emotional-affective component, little is known of its role in the sensory-discriminative component of pain^27,28^. However, the ACC has recently emerged as a region involved in modulating the sensory-discriminative component of pain, which suggests that the ACC is involved in sensory components in addition to emotional-affective components^29,30^. In the present study, similar to our previous finding in which CXCR3 inhibition in the ACC ameliorated pain^10^, pharmacological inhibition of NR2B or knockdown of PSD-95 attenuated peripheral nerve injury-induced NP, suggesting an important role of the ACC in pain modulation. This finding was also supported by recent findings that under chronic pain conditions, the descending facilitation pathways are activated from the ACC to the spinal dorsal horn, thus enhancing pain transmission via tonic facilitation^31,32^. However, further research is needed to determine whether the analgesic effect found in the present study was attributed to the disturbance of such pathways.

To further explore the role of NR2B in the ACC in pain modulation after CCI, we performed pharmacological inhibition of NR2B in the ACC by microinjections. The behavioral results showed that inhibiting NR2B attenuated behavioral hyperalgesia induced by CCI, indicating a critical role of NR2B in pain perception in the ACC. However, after antagonizing NR2B with Ro 25-6981, thermal perception seemed more sensitive than mechanical perception. While perturbation of the NR2B-PSD-95 interaction in the spinal cord reportedly attenuates thermal and mechanical pain hypersensitivity induced by either nerve injury, malignant tumors, or diabetes, no obvious difference was found between the two types of nociception with those manipulations, suggesting that spinal NR2B and PSD-95 play similar roles in this two noxious stimuli-induced pain hypersensitivity^22,33,34^. Other evidence demonstrates that optogenetic activation of locus ceruleus neurons, which receive projections from the ACC, evokes bidirectional changes in thermal nociception in rats, suggesting that the thermal-dominant neuronal circuit between the ACC and other brain regions may contribute to this discrepancy^35,36^.

**Figure 6:**
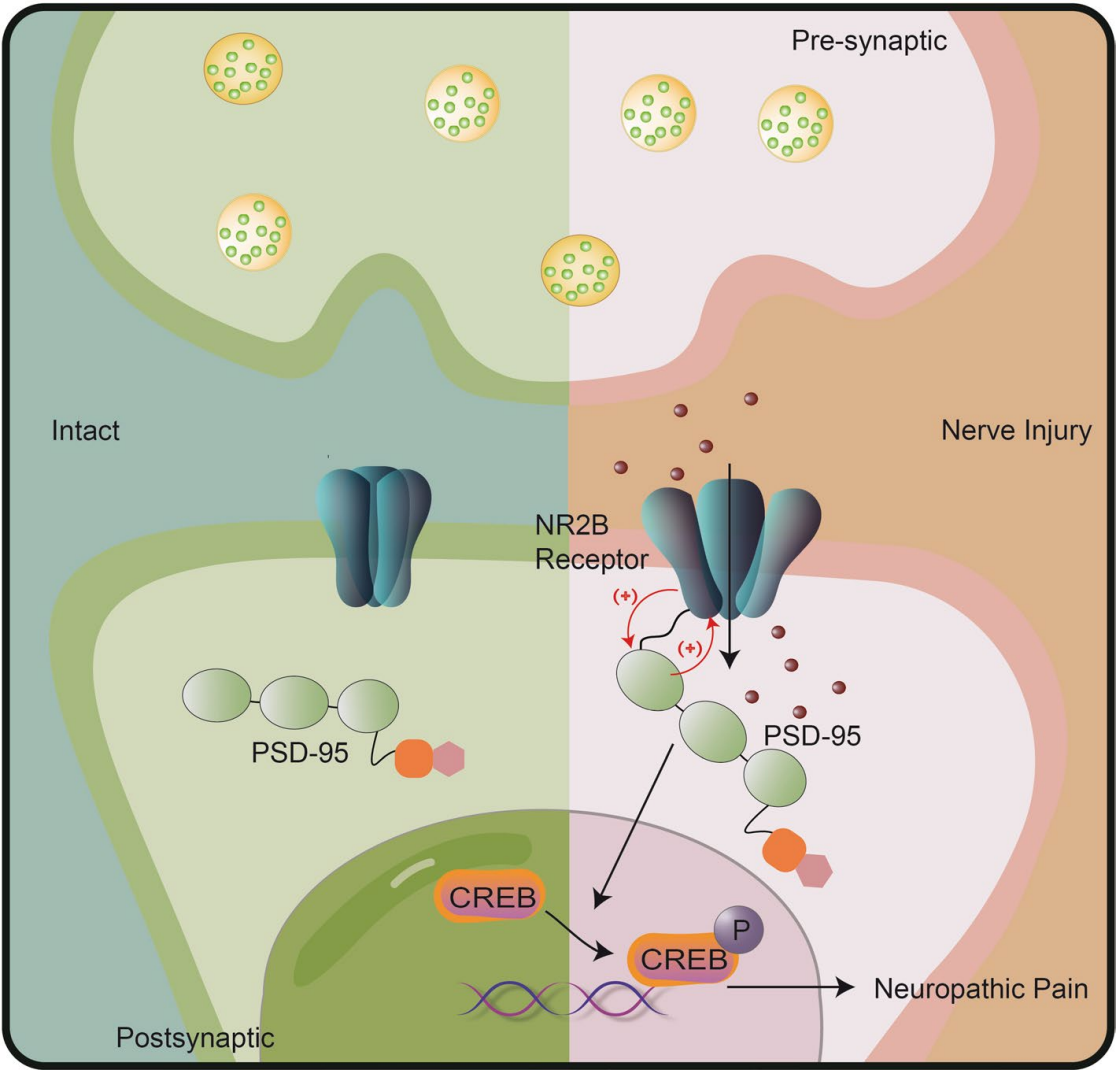
A schematic presentation of PSD-95 in the anterior cingulate cortex contributes to neuropathic pain by interdependent activation with NR2B. The material of the schematic diagram is provided by Figdraw.

PSD-95 is a scaffolding protein located on the dendritic spines of post-glutamatergic neurons that interact with NR2A or NR2B receptor subunits and plays an important role in the clustering and synaptic targeting of AMPA and NMDA receptors^37^. In the ACC, PSD-95 has been implicated in several neurological diseases^38,39^. As one of the primary neuronal cell types, pyramidal neurons express NMDA receptors on their membranes, and the NMDA receptors are involved in the modulation of various somatic and visceral noxious stimuli^40,41^. In the current study, we found that PSD-95 was well co-localized with NR2B, and CCI induced bilateral upregulation of PSD-95 and NR2B in the ACC, contrary to unilateral changes in the spinal cord^40^. This difference might be due to the crossover of the spinothalamic tracts in the anterior white commissure and the afferent projections from the contralateral pain transmitting area to the ACC^42,43^. To explore the role of PSD-95 in the ACC in pain modulation, AS-ODN was administered to knock down its expression. Behavioral tests revealed that PSD-95 knockdown attenuated hyperalgesia, while western blotting showed a downregulated NR2B expression. This reveals a PSD-95-dependent upregulation of NR2B in CCI-induced NP. To further reveal the molecular mechanisms underlying PSD-95 modulating NP, we detected the activation of CREB induced by CCI. The results showed that PSD-95 knockdown did not change the expression of CREB, but inhibited its phosphorylation, indicating that CREB might be the downstream molecule of PSD-95. Combined with the downregulation of NR2B after PSD-95 knockdown, it can be inferred that PSD-95 and NR2B are interdependently activated after CCI. A schematic presentation is shown in Fig. 6. The limitations of our study are that we did not explore if the NP-modulating mechanisms of PSD-95 and NR2B in the ACC are related to the disruption of descending facilitation pathways.

## Conclusions

The present study demonstrated that PSD-95 in the ACC plays a critical role in the modulation of NP by interdependent activation of NR2B, suggesting potential therapeutic implications for the treatment of neuropathic pain.

## Materials and methods

### Ethics

All animal experiments complied with the ARRIVE guidelines and internationally accredited guidelines and ethical regulations on animal research^44^. The protocol was approved by the Research Ethics Committee of the First Affiliated Hospital at Zhejiang University. All efforts were made to minimize the suffering and the number of animals used.

### Animals

Male Sprague–Dawley rats (weight, 220 ± 20 g) were obtained from the Experimental Animal Center of the Zhejiang Academy of Medical Sciences. Rats were maintained at 3–4 per cage at a temperature of 24 °C ± 2 °C in a 12-h light/dark cycle, with food and water ad libitum. The rats were randomly allocated to the groups. Each group contained six rats for behavioral tests and three or four rats for molecular studies. The rats received subcutaneous injections of 80,000 U of penicillin after each surgical procedure to prevent infection.

### Study design

First, naïve rats were used to explore the cellular distribution of NR2B and PSD-95 in the ACC. Second, the rats were randomly allocated to sham and CCI groups. Behavioral tests (TL and MT) were performed before and 7 days after the CCI to confirm the establishment of neuropathic pain, and western blotting was used to detect the expression changes of the target molecules (NR2B, PSD-95, CREB, and pCREB) after the CCI. Third, CCI was performed 7 days after ACC catheterization, and Ro 25-6981 was administered on days 5, 6, and 7 after CCI to investigate the role of NR2B in the neuropathic pain. TL and MT were measured 2 h after ACC administration, and western blotting was used to detect the change in expression of PSD-95 after administering Ro 25-6981. Fourth, CCI surgery was performed 7 days after ACC catheterization, and AS-ODN was injected on days 5, 6, and 7 after the CCI to investigate the role of PSD-95 in neuropathic pain. TL and MT were measured 2 h after every ACC administration and 1 day after the last administration, and western blotting was used to detect changes in the expression of the target molecules (NR2B, PSD-95, CREB, and pCREB) after the application of AS-ODN. Finally, strength of grasp of the front paws was tested on day 7 (Ro 25-6981) or 8 (AS-ODN) to rule out locomotive ability impairment after ACC catheterization and manipulation.

### Induction of NP

After anesthetization with isoflurane, the rat’s left sciatic nerve was exposed through blunt dissection of the middle thigh. In the CCI group, the sciatic nerve was isolated and ligated using three 4/0 chromic catgut sutures (Pudong Jinghuan Co. Ltd., Shanghai, China) at a spacing of 1 mm, as described in our previous studies and in a study by Bennett and Xie^45–47^. Then, the muscles and skin were closed with sutures layer-by-layer. In the sham group, the same procedure was performed without ligation.

### Pain behavioral tests

Rats were acclimated for 2 days (30 min a day) in a plastic box (12 cm × 15 cm × 22 cm) on an elevated platform before the experiment, and the experimenter was blinded to the treatment received by the rats.

*Thermal latency (TL)* A noxious light produced by a radiant thermal stimulator (Model 336; IITC/life Science) was shed on the plantar surface of the rat’s hind paw to determine the TL, as described by Hargreaves et al.^48^. The time of quick withdrawal or licking of the paw was taken as the TL. To avoid burn injuries, stimulation was terminated if no positive response was observed 20 s after initiation. Each rat received three trials with an interval of 5 min, and the average value was taken as the final TL.

*Mechanical threshold (MT)* The electronic von Frey Anesthesiometer (Model 2390; IITC/life Science, Victory Blvd Woodland Hills, CA, USA) was used to determine the paw mechanical withdrawal threshold (MT). The plantar surface was stimulated with an increasing force until a quick withdrawal or licking of the paw was noted, and the magnitude of the corresponding force was recorded automatically. Each rat received three trials with an interval of 5 min, and the average value was considered the final MT.

### ACC catheterization and drug administration

ACC catheterization and drug administration were performed according to our previous report^10^. Rats were anesthetized with pentobarbital sodium (60 mg/kg), after which the head was fixed on a stereotactic apparatus to expose the skull. Two holes were drilled on each side (Bregma forward, 1.7 mm; lateral, 0.6 mm) and a trocar (Shenzhen Ruiwode Life Science and Technology Co., Ltd, Guangdong, China) was inserted. Two small screws were superficially installed in the occipital bone to fix the catheterization with dental methyl methacrylate. CCI was performed 7 days after the catheterization. Ro 25-6981 (Med Chem Express, USA) was dissolved in 10% DMSO at a concentration of 2.5 μg/μL. PSD-95 AS-ODN (Sangon Biotech Co. Ltd., Shanghai, China) was mixed with polyethyleneimine (PEI; ExGen 500; Fermentas, Waltham, MA, USA) according to the manufacturer’s protocol. Drugs or vehicle (0.5 μL on each side) were bilaterally injected into the ACC on days 5, 6, and 7 after CCI surgery.

### Immunofluorescence assay

After deep anesthetization, rats were transcardially perfused with saline, followed by 4% paraformaldehyde. After perfusion, the ACC was harvested, post-fixed with 4% paraformaldehyde for 48 h, and then dehydrated with 30% sucrose for 3 days at 4 °C. Subsequently, the ACC was transversely cut into slices with a thickness of 30 µm. The sections were blocked with 10% sheep or donkey serum for 2 h at room temperature and incubated with the following primary antibodies for 48 h at 4 °C: goat-anti-Iba1 (1:200, Abcam), mouse-anti-GFAP (1:500, Cell Signaling Technology), mouse-anti-NeuN (1:2000, Abcam), rabbit-anti-NR2B (1:800, Cell Signaling Technology), and mouse-anti-PSD-95 (1:100, Abcam). The sections were then rinsed with PBS and incubated with the appropriate secondary antibodies in the dark for 2 h at room temperature. Finally, the sections were examined under a fluorescence microscope (FV3000; Olympus, Japan).

### Western blot analysis

Rats were deeply anesthetized using pentobarbital sodium and decapitated, and then the brain was harvested, and the ACC was divided into left and right sides for protein analysis, as described in our previous publication^10^. The protein samples were separated by SDS-PAGE (Sodium dodecyl-sulfate polyacrylamide gel electrophoresis) and transferred to a polyvinylidene difluoride membrane. In most experiments, membranes were cut according to protein marker and blocked in 5% skimmed milk at room temperature for 1 h. Then, the fragments were incubated with the corresponding primary antibodies for 24 h at 4 °C: rabbit-anti-NR2B (1:500, Abcam, Cambridge, UK), rabbit-anti-PSD-95 (1:2000, Cell Signaling Technology, Danvers, MA, USA), rabbit-anti-CREB (1:1000, Cell Signaling Technology), rabbit-anti-pCREB (1:1000, Cell Signaling Technology), and mouse-anti-GAPDH (1:10,000, Proteintech, Rosemont, IL, USA). The membrane was incubated in horseradish peroxidase-conjugated secondary antibody for 2 h at 23 °C room temperature after TBST washing. The ChemiDoc MP System (Bio-Rad, Hercules, CA, USA) was used to detect immune complex bands.

### Locomotive ability

To rule out locomotive ability impairment, the strength of grasp of the front paws was measured using a YLS-13A grasp tester (Jinan Yiyan Science Co. Ltd., Shandong, China). Briefly, the grasp force tester was placed horizontally on the ground, and the rats were placed on the plate with the front paws tightly grasping the steel wire. At this point, the rat was pulled back with increasing force until the front paw came off the steel wire. The strength of the grasp was automatically recorded. Measurements for each rat were obtained three times at 5-min intervals, and the average value was taken as the final grasp strength.

### Statistical analysis

All data are expressed as mean ± standard error of the mean (SEM) and analyzed using GraphPad Prism 8.0 (GraphPad, San Diego, CA, USA). Comparisons of behavioral data between the two groups were performed using independent *t*-tests. Behavioral differences across time points were analyzed using a two-way analysis of variance (ANOVA) followed by Bonferroni post-hoc testing. Western blot data were compared using one-way ANOVA (among the three groups) or independent *t-*tests (between the two groups). Statistical significance was set at *P* < 0.05.

## Supplementary Information

Supplementary Information.

## Data Availability

All data generated or analyzed during this study are included in this published article. The datasets generated during and/or analyzed during the current study are available from the corresponding author on reasonable request.
