# Supplementary material for: PSD-95 in the anterior cingulate cortex contributes to neuropathic pain by interdependent activation with NR2B
Source: Sci Rep. 2022 Oct 12;12:17114. doi: 10.1038/s41598-022-21488-7 (PMC9556829; doi:10.1038/s41598-022-21488-7)
Supplement: Supplementary file 1 — Supplementary Information. [file 41598_2022_21488_MOESM1_ESM.pdf]

PSD-95 in the anterior cingulate cortex contributes to neuropathic pain by interdependent activation with NR2B

Ang Li, Chang-Jun Huang, Kai-Peng Gu, Yan Huang, Ya-Qin Huang, Hui Zhang, Jia-Piao Lin, Yu-Fan Liu, Yan Yang, and Yong-Xing Yao*

List of Supplementary Information

1, Specificity of antibody to the antigen

2, Original blots of cropped images

Fig S2. Original blots for Figure 2E.

Fig S2. Original blots for Figure 2F.

Fig S2. Original blots for Figure 2G.

Fig S2. Original blots for Figure 2H.

Fig S3. Original blots for Figure 3F, G, H, I.

Fig S4. Original blots for Figure 4E.

Supplementary Information

1, Specificity of antibody to the antigen

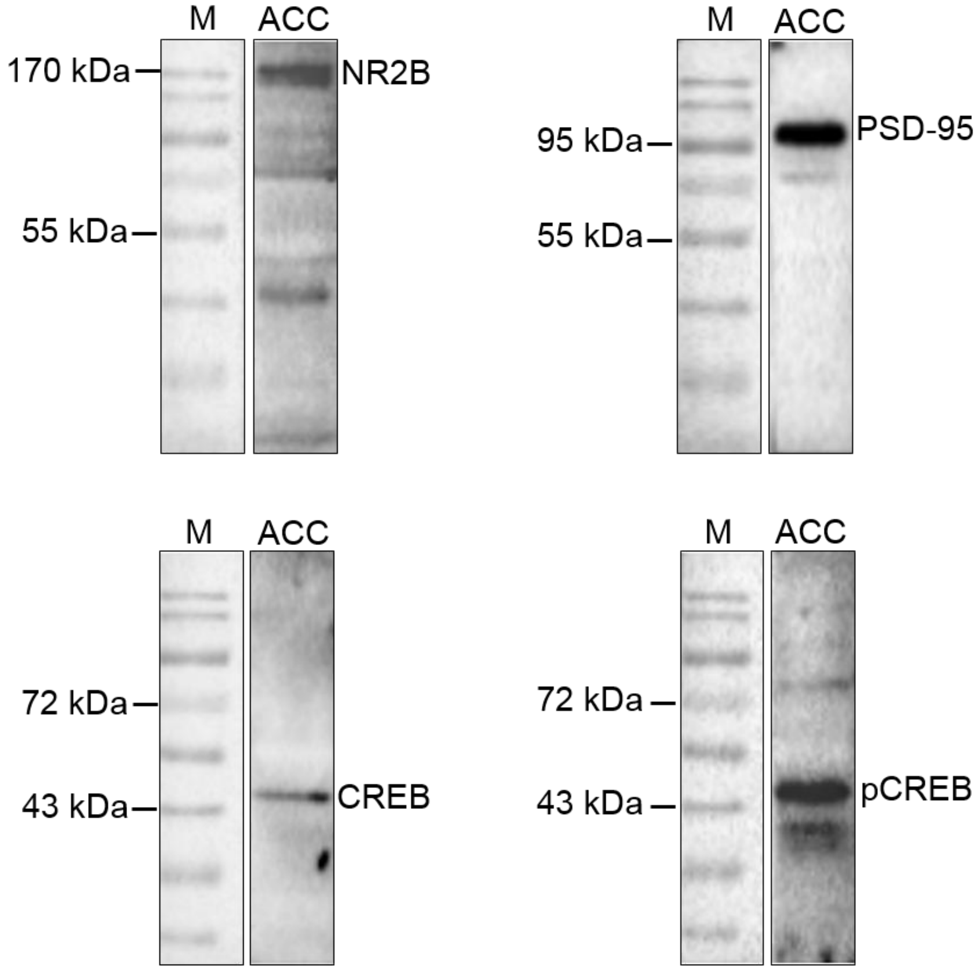

M, Prestained Protein Marker; ACC, Anterior Cingulate Cortex

Prestained Protein Marker (Thermo Fisher, 26616)

Rabbit-anti-NR2B (Abcam, ab254356)

Rabbit-anti-PSD-95 (CST, 3409)

Rabbit-anti-CREB (CST, 9197)

Rabbit-anti-pCREB (CST, 9198)

2, Original blots of cropped images

Fig S2. Original blots for Figure 2E (n = 4).

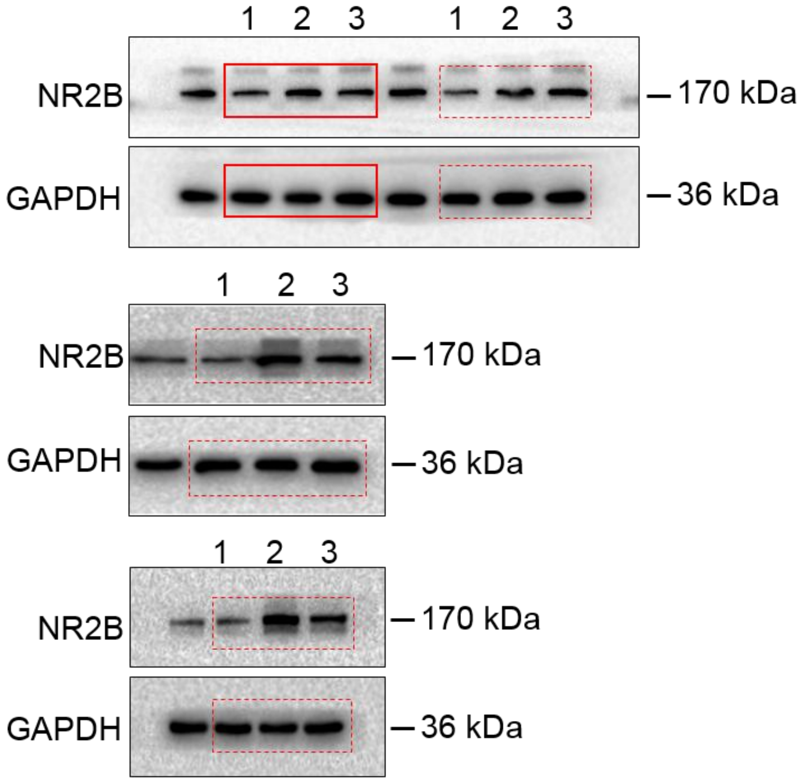

1, Sham; 2, Ipsi.; 3, Contr.

Fig S2. Original blots for Figure 2F (n = 4).

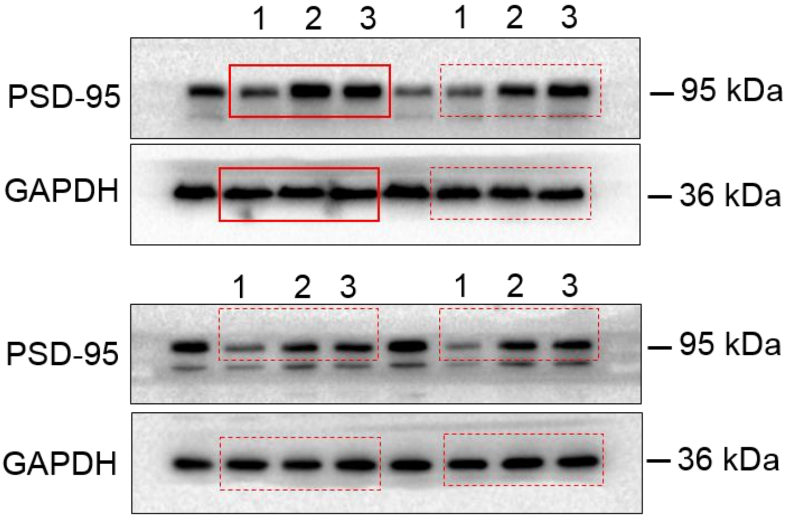

1, Sham; 2, Ipsi.; 3, Contr.

Fig S2. Original blots for Figure 2G (n = 4).

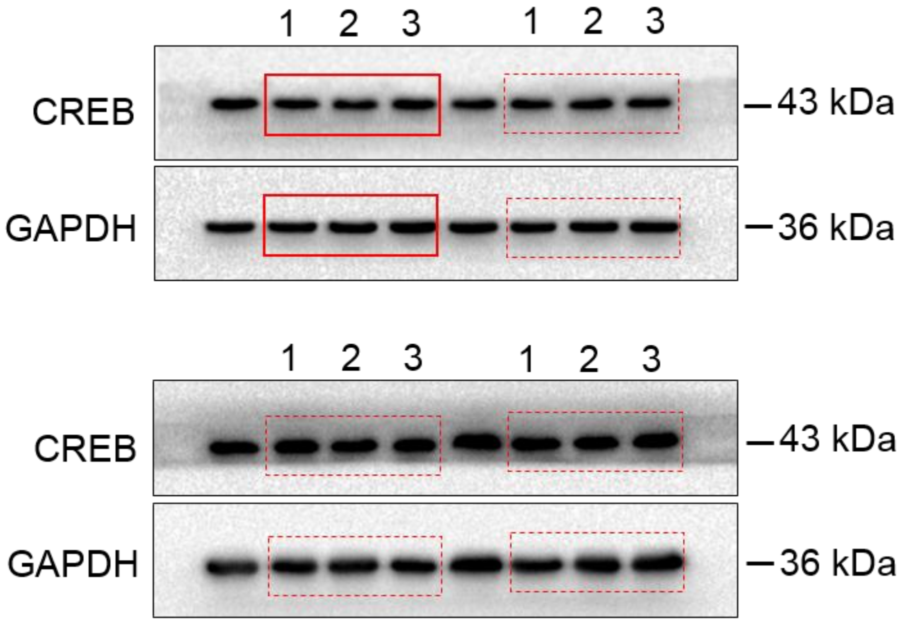

1, Sham; 2, Ipsi.; 3, Contr.

Fig S2. Original blots for Figure 2H (n = 4).

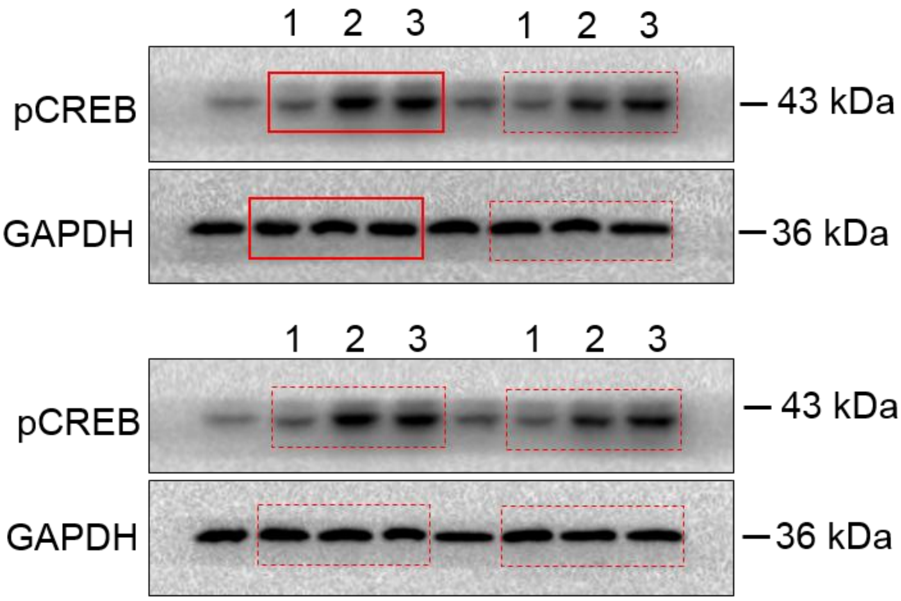

1, Sham; 2, Ipsi.; 3, Contr.

Fig S3. Original blots for Figure 3F, G, H, I (n = 3).

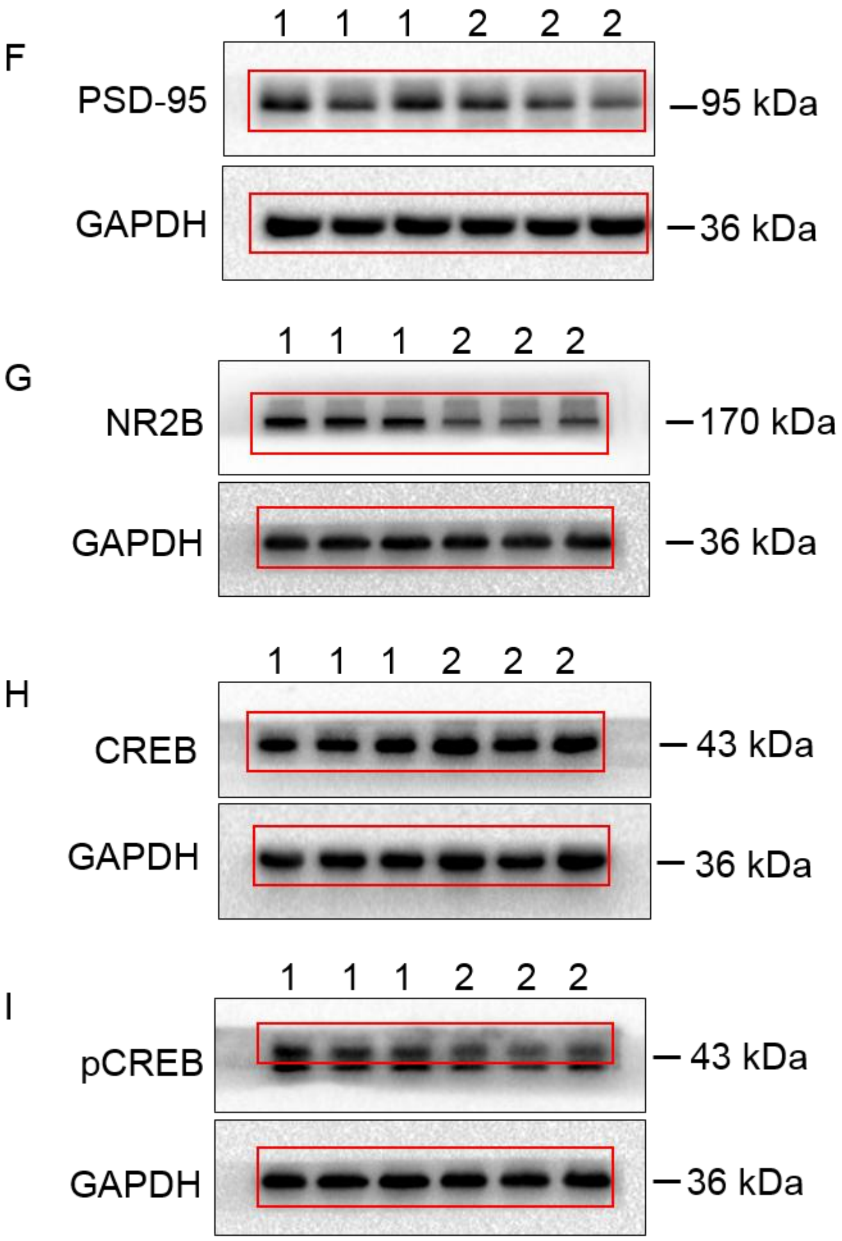

1, CCI+PEI; 2, CCI+AS-ODN

Fig S4. Original blots for Figure 4E (n = 3).

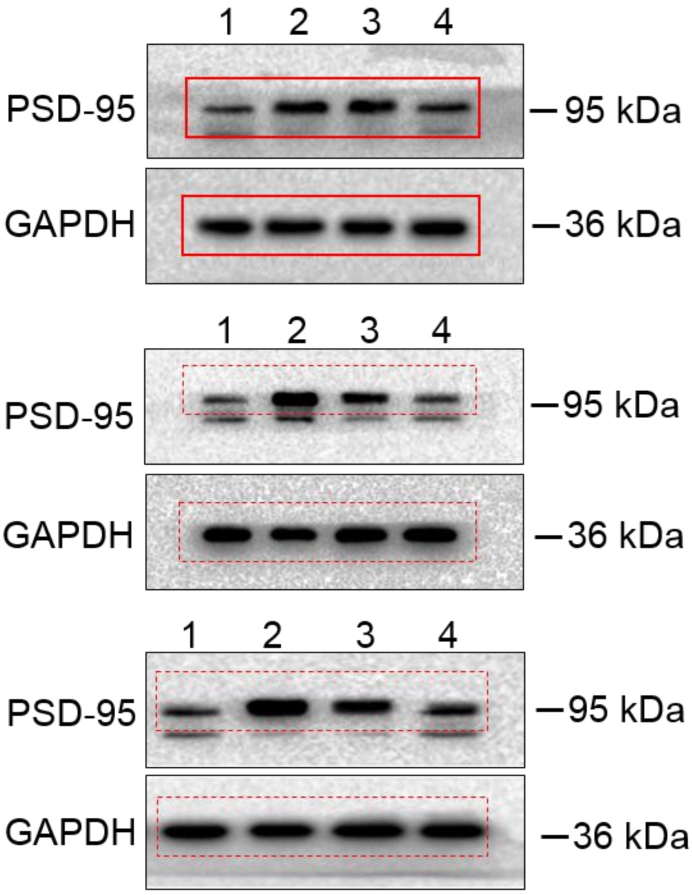

1, Sham; 2, CCl; 3, CCl+DMSO; 4, CCl+Ro 25-6981
